# A longitudinal comparison of symptom-distress and person-centeredness between a fast-track programme for cardiac surgery and conventional care

**DOI:** 10.1371/journal.pone.0343100

**Published:** 2026-03-04

**Authors:** Marita Dalvindt, Shahab Nozohoor, Anna Forsberg, Martina Lundmark

**Affiliations:** 1 Institute of Health Sciences at Lund University, Lund, Sweden; 2 Ystad Hospital, Hospital Management, Region Skåne, Sweden; 3 Department of Cardiothoracic Surgery, Lund University, Skåne University Hospital, Lund, Sweden; James Cook University Hospital, UNITED KINGDOM OF GREAT BRITAIN AND NORTHERN IRELAND

## Abstract

**Aim and design:**

Impaired recovery in the post-operative phase after cardiac surgery predicts long-term recovery, making it important to identify and treat symptoms immediately. A fast-track programme after cardiac surgery was implemented. When developing such programmes, the patients’ perspective of perioperative care is of major importance. This is a non-randomized longitudinal observational study in which symptom distress, perceived person-centeredness and pain level were prospectively compared after cardiac surgery. The aim of this non-randomized longitudinal observational study is to prospectively compare symptom-distress, perceived person-centeredness and pain level after cardiac surgery between a fast-track group and those undergoing conventional postoperative care.

**Methods:**

Symptom distress was explored and compared between a fast-track group and those undergoing conventional postoperative care. A total of 149 participants, 75% men and 25% women, with a mean age of 67 years (SD 9.9 years), were included. The control group comprised 117 patients and the fast-track group 31. Symptom distress was assessed by the Post-Operative Recovery Profile Instrument, person-centeredness by the Being Taken Seriously Questionnaire, while a Numerical Rating Scale was employed to measure self-rated pain.

**Results:**

Regarding symptom distress, the control group felt significantly (p = .041) more restricted in physical activity in the thoracic intensive care unit (TICU) compared to the fast-track group. However, no differences in symptom distress between the groups were reported in the surgical ward. Longitudinally, an increase in symptom distress was found in the TICU, followed by improvements at the surgical ward. The fast-track group demonstrated lower pain ratings at all time points compared to the control group. The pain intensity score was relatively high during the first six hours after extubation. There was no difference between the control group and the fast-track group regarding their experiences of receiving person-centred care at either of the two time points.

**Conclusions:**

A fast-track programme is equivalent to conventional care in terms of perceived symptom-distress and person-centeredness and also reduces the duration of ICU care.

**Impact and patient contribution:**

Detailed information regarding symptom distress could be used as an evidence-based foundation for symptom management support. The results contribute to an understanding of expected symptom patterns during cardiac surgery, which might increase self-efficacy when used to inform and educate patients perioperatively.

## Introduction

Enhanced Recovery After Surgery (ERAS^®^) is an international multimodal and multi-professional concept aimed at standardizing perioperative care by developing evidence-based programmes [1]. Research on ERAS^®^ programme development has traditionally focused on medical parameters such as mortality, morbidity, surgical complications and optimizing medical care [2]. However, of high importance when developing such programmes is the patients’ perspective of perioperative care, which impacts rehabilitation and thus forms the rationale behind this study. Standardizing perioperative care will minimize complications, as well as increasing efficiency and patient satisfaction. Several protocols have been developed in a variety of disciplines [1].

Cardiac surgery involves a multitude of pathologies, comorbidities and surgical approaches that hamper the development of a universal protocol encompassing the overall perioperative care of these patients [3]. The ERAS^®^ Cardiac Society (ERCS) has recently developed the first consensus-based recommendations for a standardized protocol after cardiac surgery, based on a review of meta-analyses, randomized clinical trials and large non-randomized studies [4]. The consensus statements are divided into preoperative, intraoperative and postoperative strategies. However, studies indicate a lack of high-quality data on the holistic approaches employed, thus broad-based multidisciplinary approaches and further efforts are necessary [4].

The prevalence of post-operative symptoms after cardiac surgery affects patients’ health-related quality of life (HRQoL) [2,5]. As symptoms are subjective, they can only be assessed by the patient, who represents the illness perspective. The first step in generating an ERAS^®^ Cardiac Surgery programme is to identify targets of interest and acquire knowledge of postsurgical events and complications [6], on which this study focused.

Many centres have implemented fast-track programmes, which share the common goal of facilitating early extubation and reducing the intensive care unit stay [6]. Systematic reviews of these fast-track pathways have revealed shorter times on the ventilator and in the intensive care unit as well as the fact that fast-track pathways are safe for use in low-risk patients [7,8]. While incorporating these fast-track principles, the ERCS programme expands the concept by designing comprehensive guidelines for the entire perioperative period [6].

Patients recovering from cardiac surgery experience numerous symptoms due to the surgery and medications, which affect their overall HRQoL [2,5]. A study following 120 patients after cardiac surgery demonstrated that impaired recovery during the first post-operative days predicted impaired recovery three months after surgery [9]. This indicates the importance of exploring experienced symptoms and symptom-distress immediately post-surgery to enable early interventions, which might enhance the long-term recovery process.

To optimize surgical outcome and provide person-centred care it is vital to integrate the patient perspective of perioperative care and recovery after cardiac surgery [2]. This is a novel step for contributing knowledge about a more holistic ERCS-programme based on patient experiences and Patient-Reported Outcome Measurement (PROM).

### Aim

The aim of this study was:

To prospectively compare symptom-distress, perceived person-centeredness and pain level after cardiac surgery between a fast-track group and those undergoing conventional postoperative care.

### Method

This study conforms to the checklist developed by Strengthening the Reporting of OBservational studies in Epidemiology (STROBE) and has a non-randomized, longitudinal observational design. The study setting was a university hospital, a tertiary referral cardiothoracic centre with the largest patient volume in the country. All adult patients accepted for cardiac surgery who met the inclusion criteria were consecutively invited to participate on admission to the surgical ward. For practical reasons, the patients had to be admitted to the surgical ward pre-operatively.

Inclusion criteria were adult patients (≥ 18 years) scheduled for cardiac surgery and fluent in the local language. Additional criteria for inclusion as a fast-track patient are described in Box 1. The perioperative and intermediary care of the fast-track patients are reported in Box 2. Exclusion criteria, irrespective of group, comprised of being scheduled for aortic surgery, heart or lung transplant recipients, transcatheter aortic valve implantation, dependence on mechanical support and not mentally lucid. An anaesthetist determined whether patients would be admitted to the fast-track unit or the conventional TICU after surgery, based on both medical and logistical considerations. This decision was typically made preoperatively but could be revised intraoperatively or postoperatively depending on clinical or logistical factors. Twenty-one patients declined participation. Nine additional patients were also eligible but not asked to participate due to logistic reasons, e.g., lack of time on the part of the staff who recruited the patients. The control group included 117 and the fast-track group 31 patients.

**Box 1. Specific criteria for patients selected for the fast-track programme**

- Expected uncomplicated post-operative course
- Patients scheduled for aortic valve replacement or Coronary Artery By-pass Grafting (CABG)
- Ejection Fraction (EF) ≥ 40%
- eGFR ≥ 50 (creatinine ≤ 130 if eGFR is not available)
- Patients not treated for Chronic Obstructive Disease nor Diabetes Type I
- Age ≤ 80 years old
- Body Mass Index (BMI) 19–35
- Normal right ventricular function
- PA-pressure ≤ 50 mmHg, estimated by echocardiography

**Box 2. The perioperative and intermediary care provided to the fast-track group.**

*Perioperative care*

- Pre-medication with T. Targiniq 10 alt 20 mg.
- Sedation with Fentanyl, Propofol, Sevoflurane, Remifentanil and Esketamine. Avoid Midazolam by induction and high doses of Esketamine (> 0,5 mg/kg).
- When terminating surgery, the following were administered; Esketamine 10−15 mg, Paracetamol 1 g, Ondansetron 4 mg, Betapred 4 mg and Fentanyl 0.2–0.3 mg.
- Consider parasternal blockade
- Avoid hypothermia

*Post-operative care in the Thoracic Intensive Care Unit (TICU)*

- A goal of extubation in the TICU within two hours and transfer to intermediary care two-four hours after extubation.
- Analgesics, antibiotics, monitoring and post-operative care were the same as standard care except for sedatives, which were terminated immediately after arrival at the TICU.
- Mobilization as soon as possible after extubation
- Transfer to intermediary care with central lines and thoracic chest-tube drainage

*Intermediary care*

Criteria for transfer to intermediary care from the TICU were; RLS 1−2, saturation 90−95%, ph 7.3-7.5, low-dose of Noradrenaline or Nitroglycerin, MAP > 65 mm Hg, Hb > 80 g/L, bleeding from thoracic chest-tubes < 100 ml/h, NRS < 5. Post-operative care in the intermediary unit was provided by nurses and healthcare professionals usually employed in the surgical ward. Special education prior to the intervention was provided regarding vasoactive drugs, artery line and basic intensive care knowledge. A maximum of three patients received care in the unit at the same time. If stable, patients in the fast-track programme were transferred from the intermediary unit to an ordinary surgical ward the day after cardiac surgery, where they received standard post-operative care. Comparisons were made between the fast-track group and those undergoing conventional postoperative treatment. Those in the fast-track group were assigned a specific code distinguishing them from the control group, whose members comprised the remaining patients undergoing cardiac surgery who did not meet the fast-track criteria.

### The instruments used

Symptom-distress was measured by two instruments, the Post-Operative Recovery Profile (PRP) and Numerical Rating Scale (NRS). The PRP assesses self-reported recovery longitudinally and is based on a concept analysis concerning recovery after surgery [10]. The instrument includes 19 items based on the theoretical framework consisting of five dimensions: physical symptoms, physical functions, psychological, social and activity [11]. A total sum-score of 19 points can be calculated and converted to a global level of recovery scale. The level of recovery is based on the number of ‘none’ responses, where 19 (out of 19) ‘none’ responses indicate fully recovered, continuing with a descending gradient down to <7 ‘none’ responses, which equates with not recovered at all [12]. The instrument was only used to measure symptom-distress, which was self-rated by the following responses on a four-point scale, i.e., none, mild, moderate and severe. The Swedish version of the instrument has been tested for validity and reliability and demonstrates high content validity, while a vast majority of the items showed a high level of intra-patient reliability [12].

In addition to the original instrument, nine symptoms specific to thoracic surgery suggested by an expert group of thoracic intensive care nurses with extensive clinical experience were added at the bottom of the list. When used on the digital tablet, the additional symptoms were clearly outlined and coded as not belonging to the original instrument. Therefore, no modifications were made to the original scale and no total sum-score that included the additional symptoms was used.

A Numerical Rating Scale (NRS) measured self-rated pain intensity as it is applicable in most settings [13]. The participants verbally assessed pain intensity by selecting a number between 0–10, where 0 equals no pain and 10 equals worst possible pain. Pain assessment was performed with focus on thoracic pain, although the pain experienced could be due to the surgical incision, thoracic drainage or fear and anxiety.

### Being Taken Seriously Questionnaire (BTSQ)

Person-centeredness was measured using the psychometrically tested eight-item Being Taken Seriously Questionnaire (BTSQ), which was developed in Swedish and has good results regarding validity and reliability in the study context [14]. The BTSQ has a six-grade Likert-scale. However, based on clinical experience and pilot testing for use in the TICU, the six-grade scale was modified to a three-grade scale with the following alternatives: “No, I don´t agree at all”, “I partly agree” or “Yes, I agree completely”. The total sum-score for the eight**-**item, three-grade Likert-scale was 24 points.

### Data collection

Data were collected from October 2020 until May 2021 with a short break from January-March due to the Covid-19 pandemic. Eligible patients were invited to participate by a nurse at the surgical ward. Written and oral information was provided, and the patients signed their informed consent. They were informed that they could withdraw at any time without consequences. A pre-decided code was assigned to each patient and a Case Report Form (CRF) attached to the patients’ medical records. The CRF followed the patient during the hospital stay with medical staff in the TICU and surgical ward documenting selected variables. In addition to the CRF, data collection was performed digitally on a tablet provided by the staff at different time-points for each instrument.

The PRP was completed pre-operatively at baseline, in the TICU 12–24 hours after extubation and at the surgical ward two days after post-operative readmission. The BTSQ was completed at discharge from the TICU and at discharge from the surgical ward. Before surgery, the participants self-rated possible ischemic pain using the NRS. Postoperatively, they rated thoracic pain at the TICU 30 minutes, two and six hours after extubation, as well as at the surgical ward two days after readmission. At the same time-points, if veins had been removed from a leg (as part of the surgical procedure) to be used for CABG, they self-rated leg pain.

### Statistics

SPSS Statistics 25 was used for the analysis [15]. In line with the mostly ordinal data, we used non-parametric methods and presented the findings with medians (IQR: Q1-Q3). The whole sample was dichotomized into two age-groups, ≤ 69 years or ≥ 70. The choice of cut-off was based on the median age of the whole sample, which was 69 years.

The Chi Square or Mann Whitney *U* tested hypothesis of differences between two un-paired groups, i.e., two different age groups, women vs men, and the fast-track vs control group. Spearman’s rho tested relationships between different ordinal variables. Values of p < 0.05 (two-tailed) were considered statistically significant.

Wilcoxon Signed Rank Test was used to longitudinally explore changes in symptom-distress and person-centeredness. Five symptoms from the PRP-instrument were chosen to explore associations between symptom distress and time on the ventilator as well as time in the TICU. The symptoms analysed were fatigue, muscular weakness, anxiety, concentration difficulties and memory impairment. The choice of symptoms was based on clinical experience of the risk of cognitive and physical impairments after ventilator treatment as well as after admission to the TICU. Spearman’s Rho was used to analyse perceived symptom-distress in the whole group in relation to time on the ventilator and time in the TICU. A relationship was considered weak when rho ≤ 0.3 and strong when rho ≥ 0.7. The BTSQ-instrument sum-score was calculated.

### Ethical considerations

The Swedish Ethical Review Authority granted permission to perform this study, Dnr 2020–00988 (date of approval 2020-05-08). The CRF attached to the participants’ medical records was coded and anonymized. The information provided was kept confidentially and stored in accordance with the General Data Protection Regulation.

## Results

A total of 149 participants, 75% men (n = 112) and 25% women (n = 37) with a mean age of 67 years (SD 9.9, range 28–90 years) participated. Demographics, indications and type of cardiac surgery are presented in Table 1.

**Table 1:** Demographics of the participants. It was possible to have more than one previous disease.

Demographics	Control group N=117	Fast-track group N=31
Sex n (%)		
Female	31 (26.5)	6 (19.4)
Male	86 (73.5)	24 (80.6)
Missing		n=1
Age		
Mean (Sd)	65.8 years (10.4)	71.4 years (6.4)
ASA Classification n (%)		
ASA 2^†^	1 (0.9)	0 (0)
ASA 3^‡^	98 (83.5)	26 (82.8)
ASA 4^§^	18 (15.6)	5 (17.2)
Missing	n=9	n=2
Previous disease frequency n (%)		
Hypertension	88 (75.3)	26 (82.8)
Previous myocardial infarction	34 (28.9)	11(34.5)
Atherosclerosis heart disease	90 (77.3)	21(69)
Hyperlipidemia	32 (27.8)	7 (24.1)
Type I Diabetes	4 (3.6)	0 (0)
Type II Diabetes	31 (26.8)	12 (36.7)
Harmful use of tobacco	10 (8.2)	0 (0)
Obesity	16(13.4)	1 (4)
Type of surgery n (%)		
CABG	57 (48.7)	53.3
Aortic Valve Replacement or repair or root replacement	32 (27.4)	36.7
Mitral Valve Replacement or repair	15 (12.8)	0
CABG + Aortic Valve Replacement	7 (6.0)	10
CABG + Mitral Valve Repair	3 (2.6)	0
Mitral and Tricuspid Valve Repair or replacement	3 (2.6)	0
Missing	n=1	n=1

^†^ASA 2 = A patient with mild systemic disease.

^‡^ASA 3 = A patient with severe systemic disease.

^§^ASA 4 = A patient with severe systemic disease that is a constant threat to life.

Median ventilator time in the TICU is presented in Table 2. In line with the fast-track protocol, the median time on the ventilator differed (p = .003) between the control group (210 minutes, IQR: 150–300) and the fast-track group (150 minutes, IQR:77.5–202.5). The median total TICU time also differed (p < .001) between the control group (27 hrs, IQR:25–30) and the fast-track group (10 hrs, IQR: 9–24.8). There were no differences between the groups regarding time in the surgical ward or total hospital time, nor was this included in the fast-track protocol.

**Table 2:** Differences in time on the ventilator and stay in the TICU and the surgical ward.

Variables	Control group	Fast-track group	p-value*
Time on ventilator (minutes)			.003
Median (q_1_/q_3_)	210 (150/300)	150 (77.5/202.5)	
Total frequency (missing)	104 (14)	21 (10)	
Time in thoracic intensive care unit (hours)			≤.001
Median (q_1_/q_3_)	27 (25/30)	10 (9/24.8)	
Total frequency (missing)	113 (5)	28 (3)	
Time in surgical ward (hours)			.459
Median (q_1_/q_3_)	97 (76/141.8)	100.5 (87.3/141)	
Total frequency (missing)	108 (10)	26 (5)	
Total hospital stay after surgery (hours)			.217
Median(q_1_/q_3_)	127 (103/171)	123 (100.8/149.3)	
Total frequency (missing)	108 (10)	26 (5)	

*Refers to the Mann Whitney U-test.

### Comparison between the control and the fast-track group (i)

The ten most common symptoms in the TICU, namely pain, fatigue, dependent on help from others, restrictions in physical activity, muscular weakness, sleep difficulties, dizziness, difficulties managing personal hygiene, changed appetite and throat discomfort, were further analysed to explore possible differences in frequency between the control and the fast-track group. No significant differences between the groups could be found in the TICU. However, in the surgical ward a higher proportion of fatigue was observed in the control group 83.5% (p = .035).

The groups were compared regarding symptom-distress. The control group felt significantly (p = .041) more restricted in physical activity in the TICU, median 3 (IQR: 2–4) compared to the fast-track group, median 2, (IQR:1.5–3). However, no differences in symptom-distress between the groups were reported in the surgical ward.

In the control group, higher symptom-distress in the TICU compared to before surgery was demonstrated by changed median scores in the following symptoms: pain from 1 to 3 (p=≤.001), fatigue from 2 to 3 (p ≤ .001), dependent on help from others from 1 to 3 (p ≤ .001), restrictions in physical activity from 1 to 3 (p ≤ .001), muscular weakness from 1 to 2.5 (p ≤ .001), sleep difficulties from 1 to 3 (p ≤ .001), dizziness from 1 to 2 (p ≤ .001), difficulties managing personal hygiene from 1 to 2 (p ≤ .001), changed appetite from 1 to 2 (p ≤ .001), throat discomfort from 1 to 2 (p ≤ .001) and concentration difficulties from 1 to 2 (p ≤ .001). Anxiety and worry were higher pre-operatively, median 2, compared to in the TICU, median 1 (p = .009).

When analysing the change in symptom-distress from the TICU to the surgical ward in the control group, improvements indicated by a lower median score could be seen in pain from 3 to 2 (p ≤ .001), fatigue from 3 to 2 (p = .012), dependent on help from others from 3 to 2 (p ≤ .001), restrictions in physical activity from 3 to 2 (p ≤ .001), muscular weakness from 2.5 to 2 (p = .042), sleep difficulties from 3 to 2 (p ≤ .001), dizziness from 2 to 1 (p ≤ .001) and difficulties managing personal hygiene from 2 to the same median, but with different percentiles (p ≤ .001).

In the fast-track group, higher symptom-distress in the TICU compared to before surgery was indicated by increased median scores in the following symptoms: pain from 1 to 3 (p ≤ .001), fatigue from 2 to 3 (p = .003), dependent on help from others from 1 to 3 (p = .002), restrictions in physical activity from 1 to 2 (p < .004), muscular weakness from 1 to 2 (p = .005), sleep difficulties from 1 to 2 (p = .011), dizziness from 1 to 2 (p = .004), difficulties managing personal hygiene from 1 to 2 (p = .001), changed appetite from 1 to 2 (p = .001) and throat discomfort from 1 to 2 (p = .026).

When analysing the change in symptom-distress from the TICU to the surgical ward in the fast-track group, improvements in median scores were found for pain from 3 to 2 (p = .001), restrictions in physical activity from 2 to the same median score, but different percentiles (p = .017) and dizziness from 2 to 1 (p = .015).

**Fig 1:**
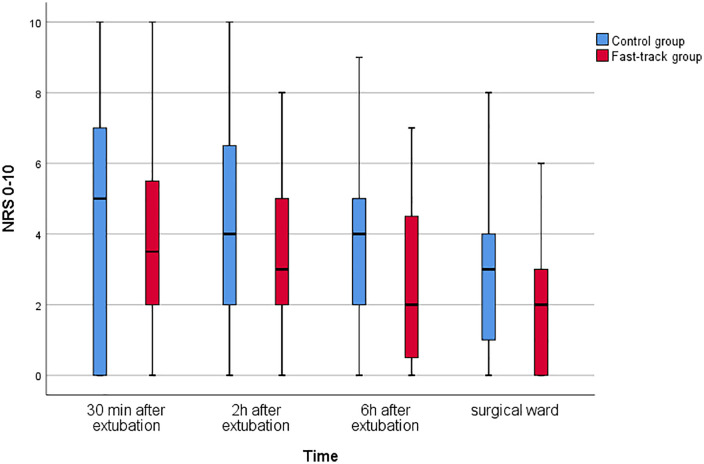
Medians of self-reported pain intensity for the control and the fast-track group at different time points after surgery.

NRS medians for the control and the fast-track group are presented in Fig 1. When comparing the medians between the groups, the fast-track group demonstrates lower NRS medians at all time-points compared to the control group. This difference was statistically significant six hours after extubation, where the fast-track group reported median NRS 2 (IQR:0–5) and the control group median NRS 4 (IQR:2–5) in the TICU (p = .04).

Longitudinal sub-group analysis of pain in the fast-track versus the control group exhibited similar pathways to the whole sample, with no significant differences over time except from 6 hours post-operatively median NRS 4 (IQR:2–5) to the surgical ward, median 3 (IQR:1–4) in the control group (p < .001). The fast-track group did not achieve statistical significance (p = .309) and had the same NRS medians at 6 hours as in the surgical ward.

### Exploration of symptom-distress (ii)

Symptom distress pre-operatively, in the TICU and in the surgical ward is presented in Tables 3–5 and provides a thorough and detailed overview of the symptoms experienced. When comparing younger patients with those over 70 years in the TICU, a significantly larger proportion of the younger group (64.7%, p = .006) reported more concentration difficulties, difficulties managing personal hygiene (65.7%, p = .001), restrictions in physical activities (61.5%, p = .001) and dependent on help from others (58.7%, p = .0034) compared to the older patients. In the surgical ward, a significantly larger proportion of the younger patients (55.2%, p = .041) reported sleep difficulties. No gender differences were revealed in the TICU, but later in the surgical ward a significantly larger proportion of male patients (69.5%, p = .008) experienced changed appetite compared to women.

**Table 3:** Differences in pre-operative symptom frequency and symptom distress between the control and the fast-track group. Only patients reporting mild to severe distress are included. The symptoms are presented in descending order starting with the most frequent symptom in the whole sample (control and fast-track group).

Pre-operative symptoms Control group + Fast track = 149	Symptom distress	Control group n=118 (%)	Fast track n= 31 (%)
**Anxiety and worry**	Mild	48 (40.7)	10 (32.3)
	Moderate	28 (23.7)	4 (12.9)
Total frequency n=98 (65.8%)	Severe	6 (5.1)	2 (6.5)
**Restrictions in everyday life**	Mild	38 (32.2)	11 (35.5)
	Moderate	27 (22.9)	5 (16.1)
Total frequency n=94 (63.1%)	Severe	11 (9.3)	2 (6.5)
**Breathlessness**	Mild	34 (28.8)	13 (41.9)
	Moderate	26 (22.0)	2 (6.5)
Total frequency n=83 (55.7%)	Severe	6 (5.1)	2 (6.5)
**Fatigue**	Mild	40 (33.9)	9 (29.0)
	Moderate	21 (17.8)	5 (16.1)
Total frequency n=80 (53.7%)	Severe	4 (3.4)	1 (3.2)
**Sleep difficulties**	Mild	31 (26.3)	10 (32.3)
	Moderate	16 (13.6)	2 (6.5)
Total frequency n=67 (45.0%)	Severe	7 (5.9)	1 (3.2)
**Rapid pulse or palpitations**	Mild	37 (31.4)	9 (29.0)
	Moderate	15 (12.7)	1 (3.2)
Total frequency n=64 (43.0%)	Severe	1 (0.8)	1 (3.2)
**Depression/feeling down**	Mild	40 (33.9)	2 (6.5)
	Moderate	14 (11.9)	3 (9.7)
Total frequency n=60 (40.3%)	Severe	1 (0.8)	0 (0)
**Muscular weakness**	Mild	30 (25.4)	10 (32.3)
	Moderate	10 (8.5)	5 (16.1)
Total frequency n=57 (38.3%)	Severe	2 (1.7)	0 (0)
**Impaired sex life**	Mild	24 (20.3)	5 (16.1)
	Moderate	12 (10.2)	4 (12.9)
Total frequency n=57 (38.3%)	Severe	10 (8.5)	2 (6.5)
**Restrictions in social life**	Mild	29 (24.6)	6 (19.4)
	Moderate	12 (10.2)	1 (3.2)
Total frequency n=53 (35.6%)	Severe	5 (4.2)	0 (0)
**Memory impairment**	Mild	31 (26.3)	11 (35.5)
	Moderate	4 (3.4)	1 (3.2)
Total frequency n=50 (33.6%)	Severe	2 (1.7)	1 (3.2)
**Dependent on help from others**	Mild	27 (22.9)	5 (16.1)
	Moderate	10 (8.5)	2 (6.5)
Total frequency n= 47 (31.5%)	Severe	2 (1.7)	1 (3.2)
**Concentration difficulties**	Mild	33 (28.0)	4 (12.9)
	Moderate	4 (3.4)	1 (3.2)
Total frequency n=44 (29.5%)	Severe	1 (0.8)	1 (3.2)
**Restrictions in physical activity**	Mild	23 (19.5)	6 (19.4)
	Moderate	7 (5.9)	2 (6.5)
Total frequency n=42 (28.2%)	Severe	2 (1.7)	2 (6.5)
**Dizziness**	Mild	26 (22.0)	9 (29.0)
	Moderate	4 (3.4)	2 (6.5)
Total frequency n=42 (28.2%)	Severe	1 (0.8)	0 (0)
**Feeling lonely and vulnerable**	Mild	28 (23.7)	4 (12.9)
	Moderate	8 (6.8)	0 (0)
Total frequency n=41 (27.5%)	Severe	0 (0)	1 (3.2)
**Throat discomfort**	Mild	24 (20.3)	6 (19.4)
	Moderate	8 (6.8)	1 (3.2)
Total frequency n=39 (26.2%)	Severe	0 (0)	0 (0)
**Vivid dreams or nightmares**	Mild	28 (23.7)	8 (25.8)
	Moderate	2 (1.7)	0 (0)
Total frequency n=39 (26.2%)	Severe	1 (0.8)	0 (0)
**Gastrointestinal function**	Mild	20 (16.9)	6 (19.4)
	Moderate	7 (5.9)	2 (6.5)
Total frequency n=37 (24.8%)	Severe	2 (1.7)	0 (0)
**Pain**	Mild	20 (16.9)	4 (12.9)
	Moderate	5 (4.2)	4 (12.9)
Total frequency n=34 (22.8%)	Severe	1 (0.8)	0 (0)
**Bladder function**	Mild	13 (11.0)	10 (32.3)
	Moderate	4 (3.4)	0 (0)
Total frequency n=27 (18.1%)	Severe	0 (0)	0 (0)
**Changed appetite**	Mild	18 (15.3)	3 (9.7)
	Moderate	2 (1.7)	2 (6.5)
Total frequency n=25 (16.8%)	Severe	0 (0)	0 (0)
**Difficulties comprehending the outside world**	Mild	5 (4.2)	1 (3.2)
	Moderate	3 (2.5)	1 (3.2)
Total frequency n=10 (6.7%)	Severe	0 (0)	0 (0)
**Nausea**	Mild	6 (5.1)	4 (12.9)
	Moderate	0 (0)	0 (0)
Total frequency n=10 (6.7%)	Severe	0 (0)	0 (0)
**Difficulties managing personal hygiene**	Mild	6 (5.1)	0 (0)
	Moderate	2 (1.7)	0 (0)
Total frequency n=8 (5.4%)	Severe	0 (0)	0 (0)

**Table 4:** Differences in symptom frequency and symptom-distress at the Thoracic Intensive Care Unit (TICU) between the control and the fast-track group. Only patients reporting mild to severe distress are included. The symptoms are presented in descending order starting with the most frequent symptom in the whole sample (control and fast-track group).

Thoracic Intensive Care Unit symptoms and frequency Control + Fast-track group = 109	Symptom distress	Control group n=88 (30 missing) N (%)	Fast-track group n= 21 (10 missing) N (%)
**Pain**	Mild	30 (34.1)	9 (42.9)
	Moderate	34 (38.6)	10 (47.6)
Total frequency n=98 (89.9%)	Severe	13 (14.8)	2 (9.5)
**Fatigue**	Mild	15 (17.0)	5 (23.8)
	Moderate	31 (35.2)	13 (61.9)
Total frequency n=93 (85.3%)	Severe	28 (31.8)	1 (4.8)
**Dependent on help from others**	Mild	20 (22.7)	6 (28.6)
	Moderate	25 (28.4)	5 (23.8)
Total frequency n=92 (84.4%)	Severe	30 (34.1)	6 (28.6)
**Restrictions in physical activity**	Mild	20 (23.0)	8 (38.1)
	Moderate	22 (25.3)	4 (19)
Total frequency n=91 (83.5%)	Severe	33 (37.9)	4 (19)
**Muscular weakness**	Mild	26 (29.5)	10 (47.6)
	Moderate	31 (35.2)	8 (38.1)
Total frequency n=88 (80.7%)	Severe	13 (14.8)	0 (0)
**Sleep difficulties**	Mild	20 (22.7)	6 (28.6)
	Moderate	25 (28.4)	4 (19)
Total frequency n=83 (76.1%)	Severe	23 (26.1)	5 (23.8)
**Dizziness**	Mild	31 (35.2)	6 (28.6)
	Moderate	21 (23.9)	7 (33.3)
Total frequency n=74 (67.9%)	Severe	8 (9.1)	1 (4.8)
**Difficulties managing personal hygiene**	Mild	22 (25.0)	8 (38.1)
	Moderate	18 (20.5)	3 (14.3)
Total frequency n=73 (67.0%)	Severe	20 (22.7)	2 (9.5)
**Changed appetite**	Mild	25 (28.4)	9 (42.9)
	Moderate	18 (20.5)	2 (9.5)
Total frequency n=71 (65.1%)	Severe	16 (18.2)	1 (4.8)
**Throat discomfort**	Mild	31 (35.2)	10 (47.6)
	Moderate	18 (20.5)	4 (19.0)
Total frequency n=69 (63.3%)	Severe	6 (6.8)	0 (0)
**Concentration difficulties**	Mild	19 (21.6)	6 (28.6)
	Moderate	26 (29.5)	5 (23.8)
Total frequency n=68 (62.4%)	Severe	12 (13.6)	0 (0)
**Restrictions in social life**	Mild	19 (21.6)	5 (23.8)
	Moderate	13 (14.8)	2 (9.5)
Total frequency n=66 (60.6%)	Severe	24 (27.3)	3 (14.3)
**Anxiety and worry**	Mild	21 (23.9)	6 (28.6)
	Moderate	18 (20.5)	0 (0)
Total frequency n=51 (46.8%)	Severe	4 (4.5)	2 (9.5)
**Breathlessness**	Mils	46 (52.3)	14 (66.7)
	Moderate	17 (19.3)	2 (9.5)
Total frequency n=49 (45.0%)	Severe	6 (6.8)	1 (4.8)
**Nausea**	Mild	25 (28.4)	7 (33.3)
	Moderate	10 (11.4)	2 (9.5)
Total frequency n=48 (44.0%)	Severe	4 (4.5)	0 (0)
**Memory impairment**	Mild	15 (17.0)	4 (19)
	Moderate	17 (19.3)	1 (4.8)
Total frequency n=40 (36.7%)	Severe	3 (3.4)	0 (0)
**Depression/feeling down**	Mild	14 (15.9)	6 (28.6)
	Moderate	12 (13.6)	0 (0)
Total frequency n=36 (33.0%)	Severe	4 (4.5)	0 (0)
**Feeling lonely and vulnerable**	Mild	13 (14.8)	3 (14.3)
	Moderate	6 (6.8)	0 (0)
Total frequency n=30 (27.5%)	Severe	8 (9.1)	0 (0)
**Vivid dreams or nightmares**	Mild	12 (13.6)	5 (23.8)
	Moderate	8 (9.1)	2 (9.5)
Total frequency n=30 (27.5%)	Severe	2 (2.3)	1 (4.8)
**Rapid pulse or palpitations**	Mild	17 (19.3)	4 (19.0)
	Moderate	5 (5.7)	0 (0)
Total frequency n=29 (26.6%)	Severe	3 (3.4)	0 (0)
**Numbness between the breasts**	Mild	15 (17.0)	5 (23.8)
	Moderate	6 (6.8)	1 (4.8)
Total frequency n=29 (26.6%)	Severe	2 (2.3)	0 (0)
**Gastrointestinal function**	Mild	17 (19.3)	2 (9.5)
	Moderate	4 (4.5)	3 (14.3)
Total frequency n=28 (25.7%)	Severe	2 (2.3)	0 (0)
**Bladder function**	Mild	11 (12.5)	0 (0)
	Moderate	3 (3.4)	2 (9.5)
Total frequency n=22 (20.2%)	Severe	5 (5.7)	1 (4.8)
**Numbness in the operated leg**	Mild	9 (10.2)	1 (4.8)
	Moderate	2 (2.3)	0 (0)
Total frequency n=15 (13.8%)	Severe	3 (3.4)	0 (0)
**Wound oozing from the operated leg or sternum**	Mild	7 (8.0)	5 (23.8)
	Moderate	2 (2.3)	0 (0)
Total frequency n=14 (12.8%)	Severe	0 (0)	0 (0)

**Table 5:** Differences in symptom frequency and symptom-distress in the thoracic surgical ward between the control and the fast-track group. Only patients reporting mild to severe distress are included. The symptoms are presented in descending order starting with the most frequent symptom in the whole sample (control group and fast-track group).

Surgical ward symptoms and frequency Control group + Fast track = 120	Symptom distress	Control group n=97 (21 missing) (%)	Fast-track group n= 23 (8 missing) (%)
**Fatigue**	Mild	52 (53.6)	8 (34.8)
Total frequency n=109 (90.8%)	Moderate	28 (28.9)	8 (34.8)
	Severe	11 (11.3)	2 (8.7)
**Muscular weakness**	Mild	55 (56.7)	9 (39.1)
Total frequency n=101 (84.2%)	Moderate	24 (24.7)	9 (39.1)
	Severe	3 (3.1)	1 (4.3)
**Dependent on help from others**	Mild	52 (53.6)	12 (52.2)
Total frequency n=99 (82.5%)	Moderate	20 (20.6)	6 (26.1)
	Severe	8 (8.2)	1 (4.3)
**Changed appetite**	Mild	38 (39.2)	9 (39.1)
Total frequency n=95 (79.2%)	Moderate	28 (28.9)	5 (21.7)
	Severe	12 (12.4)	3 (13.0)
**Restrictions in social life**	Mild	31 (32)	5 (21.7)
Total frequency n=91 (75.8%)	Moderate	25 (25.8)	6 (26.1)
	Severe	20 (20.6)	4 (17.4)
**Pain**	Mild	51 (52.6)	15 (65.2)
Total frequency n=90 (75.0%)	Moderate	19 (19.6)	4 (17.4)
	Severe	1 (1.0)	0 (0)
**Sleep difficulties**	Mild	39 (40.2)	7 (30.4)
Total frequency n=87 (72.5%)	Moderate	28 (28.9)	6 (26.1)
	Severe	4 (4.1)	3 (13.0)
**Throat discomfort**	Mild	43 (44.3)	13 (56.5)
Total frequency n=76 (63.3%)	Moderate	16 (16.5)	3 (13.0)
	Severe	1 (1.0)	0 (0)
**Difficulties managing personal hygiene**	Mild	45 (46.4)	12 (52.2)
Total frequency n=74 (61.7%)	Moderate	15 (15.5)	1 (4.3)
	Severe	1 (1.0)	0 (0)
**Restrictions in physical activity**	Mild	41 (42.3)	7 (30.4)
Total frequency n=72 (60.0%)	Moderate	13 (13.4)	6 (26.1)
	Severe	5 (5.2)	0 (0)
**Breathlessness**	Mild	40 (41.2)	8 (34.8)
Total frequency n=71 (59.2%)	Moderate	15 (15.5)	4 (17.4)
	Severe	4 (4.1)	0 (0)
**Gastrointestinal function**	Mild	43 (44.3)	10 (43.5)
Total frequency n=71 (59.2%)	Moderate	10 (10.3)	4 (17.4)
	Severe	4 (4.1)	0 (0)
**Anxiety and worry**	Mild	37 (38.1)	6 (26.1)
Total frequency n=62 (51.7%)	Moderate	11 (11.3)	2 (8.7)
	Severe	6 (6.2)	0 (0)
**Concentration difficulties**	Mild	35 (36.1)	5 (21.7)
Total frequency n=58 (48.3%)	Moderate	13 (13.4)	1 (4.3)
	Severe	4 (4.1)	0 (0)
**Dizziness**	Mild	31 (32.0)	7 (30.4)
Total frequency n=50 (41.7%)	Moderate	8 (8.2)	1 (4.3)
	Severe	3 (3.1)	0 (0)
**Rapid pulse or palpitations**	Mild	23 (23.7)	6 (26.1)
Total frequency n=49 (40.8%)	Moderate	13 (13.4)	3 (13.0)
	Severe	4 (4.1)	0 (0)
**Depression/feeling down**	Mild	28 (28.9)	6 (26.1)
Total frequency n=46 (38.3%)	Moderate	11 (11.3)	0 (0)
	Severe	1 (1.0)	0 (0)
**Feeling lonely and vulnerable**	Mild	24 (24.7)	3 (13.0)
Total frequency n=42 (35.0%)	Moderate	11 (11.3)	2 (8.7)
	Severe	2 (2.1)	0 (0)
**Memory impairment**	Mild	27 (27.8)	5 (21.7)
Total frequency n=40 (33.3%)	Moderate	6 (6.2)	0 (0)
	Severe	2 (2.1)	0 (0)
**Nausea**	Mild	21 (21.6)	6 (26.1)
Total frequency n=40 (33.3%)	Moderate	9 (9.3)	0 (0)
	Severe	4 (4.1)	0 (0)
**Vivid dreams or nightmares**	Mild	19 (19.6)	4 (17.4)
Total frequency n=39 (32.5%)	Moderate	11 (11.3)	1 (4.3)
	Severe	3 (3.1)	1 (4.3)
**Bladder function**	Mild	15 (15.6)	7 (30.4)
Total frequency n=31 (25.8%)	Moderate	5 (5.2)	0 (0)
	Severe	4 (4.2)	0 (0)
**Numbness between the breasts**	Mild	16 (16.5)	4 (17.4)
Total frequency n=23 (19.2%)	Moderate	3 (3.1)	0 (0)
	Severe	0 (0)	0 (0)
**Wound oozing from the operated leg or sternum**	Mild	8 (8.2)	4 (17.4)
Total frequency n=15 (12.5%)	Moderate	2 (2.1)	0 (0)
	Severe	1 (1.0)	0 (0)
**Numbness in the operated leg**	Mild	11 (11.3)	2 (8.7)
Total frequency n=13 (10.8%)	Moderate	0 (0)	0 (0)
	Severe	0 (0)	0 (0)

Weak correlations during the TICU stay were observed between time on the ventilator, fatigue (r_s_ = .212) and muscle weakness (r_s_ = .315). A weak correlation between time on the ventilator and concentration difficulties was found at the surgical ward (r_s_ = .249). There was no relationship between time spent in the TICU and the five analysed symptoms (fatigue, muscular weakness, anxiety, concentration difficulties and memory impairment), either at the TICU or surgical ward.

Before surgery 12.2% (n = 17) of 139 patients experienced ischemic pain expressed as a median NRS-score 4. Patients who experienced pre-operative anxiety and worry self-rated their post-operative experience of pain significantly higher (p = .031), median NRS 6 (Q1 4; Q3 7.5) two hours after extubation, compared to those without preoperative anxiety, median NRS 5(Q1 3; Q3 7.25).

A total of 115 patients self-assessed the BTSQ in the TICU versus 103 patients in the surgical ward. The median score was 24 (IQR:24–24) in the TICU and 24 (IQR 23–24) in the surgical ward. There was no difference between the control group and the fast-track group at either of the two time points. There were no differences in BTSQ between the TICU and the surgical ward (p = .495) nor any differences over time between the fast-track group (p = .655) and the control group (p = .592).

### Longitudinal changes in symptom-distress

A significant difference in symptom-distress from pre-operatively to the TICU was found for all symptoms except rapid pulse or palpitations, depression and gastrointestinal function, presented in Table 6. The symptom-distress increased in the TICU except for anxiety and worry, which was higher pre-operatively. The symptom-distress trajectory between the TICU and the surgical ward indicates an overall improvement in many symptoms.

**Table 6:** Longitudinal changes in symptom distress from before cardiac surgery, via the Thoracic Intensive Care Unit (TICU) to post surgery at the thoracic surgical ward.

Symptom	Before surgery	In the TICU		At the thoracic ward	
	Median (q1; q3)	Median (q1; q3)	p-value* (Pre-op to TICU)	Median (q1; q3)	p-value* (From TICU to ward)
**Nausea**	1 (1; 1)	1 (1; 2)	**.000**	1 (1; 2)	.096
**Gastrointestinal function**	1 (1; 1.5)	1 (1; 2)	.900	2 (1; 2)	**.000**
**Fatigue**	2 (1; 2)	3 (2; 4)	**.000**	2 (2;3)	**.003**
**Muscular weakness**	1 (1; 2)	2 (2; 3)	**.000**	2 (2;3)	**.050**
**Changed appetite**	1 (1; 1)	2 (1; 3)	**.000**	2 (2;3)	.070
**Sleep difficulties**	1 (1; 2)	3 (2;4)	**.000**	2 (1;3)	**.000**
**Anxiety and worry**	2 (1; 3)	1 (1;2)	**.003**	2 (1;2)	.519
**Depression/feeling down**	1 (1; 2)	1 (1;2)	.794	1 (1;2)	.563
**Restrictions in social life**	1 (1; 2)	2 (1; 3.5)	**.000**	2 (2;3)	.191
**Difficulties managing personal hygiene**	1 (1; 1)	2 (1;3)	**.000**	2 (1;2)	.**000**
**Bladder function**	1 (1; 1)	1 (1;1)	**.025**	1 (1;2)	.929
**Restrictions in physical activity**	1 (1; 2)	3 (2;4)	**.000**	2 (1;2)	**.000**
**Feeling lonely and vulnerable**	1(1;2)	1(1;2)	**.045**	1 (1;2)	.933
**Depending on help from others**	1(1;2)	3 (2;4)	**.000**	2 (2;3)	**.000**
**Concentration difficulties**	1(1;2)	2 (1;3)	**.000**	1 (1;2)	**.001**
**Memory impairment**	1(1;2)	1(1;2)	**.022**	1 (1;2)	.112
**Throat discomfort**	1(1;2)	2 (1;3)	**.000**	2 (1;2)	.201
**Vivid dreams or nightmares**	1(1;2)	1(1;2)	**.048**	1 (1;2)	.628
**Dizziness**	1(1;2)	2 (1;3)	**.000**	1 (1;2)	**.000**
**Breathlessness**	2(1;2)	1(1;2)	**.490**	2 (1;2)	.641
**Rapid pulse or palpitations**	1(1;2)	1(1;2)	.066	1 (1;2)	**.012**

* Represents Wilcoxon Signed Rank Test.

There was no difference in NRS ratings in the TICU from 30 min after extubation until 2 hours afterwards (p = .197), nor from 2 hours after extubation until 6 hours afterwards (p = .963), although a difference was found between 6 hours after extubation, median NRS 4 (IQR: 2–5) and two days post-operatively in the surgical ward, median NRS 3 (IQR:1–4, p < .001). A clinical but not significant improvement in NRS-ratings was identified over time, indicated by decreasing or the same NRS medians.

### Age and sex analysis of pain

There was a difference in NRS ratings with significantly decreasing medians (p = .039) from 30 minutes after extubation, median 5, until 2 hours afterwards in the group of men, median 4. However, in women (p = .404) the median increased from 4 to 5, indicating a clinical deterioration, although not statistically significant. In the groups of men and women, no differences in NRS ratings could be found from 2 hours after extubation until 6 hours afterwards, although both groups exhibited differences from 6 hours after extubation, median 4 and in the surgical ward, median 2 (p ≤ .001).

When comparing two age-groups, ≤ 69 or >70 years, no significant differences over time were found except from 6 hours to the surgical ward, where both groups had improved NRS ratings (≤69 from median 5–4 p < .001 and > 70 years from median 5.5 to 3 p = .045). The NRS medians in the group of older patients were lower at all time points.

## Discussion

This study contributes a systematic and thorough overview of symptom-distress and experienced person-centeredness after cardiac surgery and how these evolve over the initial post-operative phase. It could be used as an evidence-based foundation for symptom management support after cardiac surgery.

Comparisons between the fast-track and control group indicate that from a patient perspective, a fast-track programme is equivalent to conventional care in terms of experienced symptom-distress and person-centeredness. Time on the ventilator and in the TICU was significantly shorter in the fast-track compared to the control group. This was the goal of the intervention and in line with the result in the review by Wong et al. [8] when implementing a fast-track protocol. There may be several reasons why the fast-track patients did not differ to a greater extent. The surgical procedure is basically the same in the two groups with a similar impact due to the surgery and subsequent treatment. Furthermore, the fast-track protocol had just been implemented and not yet fine-tuned by the staff involved. We expect a more pronounced difference between the two groups if the study was repeated today. The Covid-19 pandemic acted as a confounding factor by removing many resources from regular postoperative care, which might have influenced both groups. From an organisational perspective, no extra staff was allocated to the fast-track patients.

Most of the findings from the exploration of symptom-distress were clinically reasonable and thus expected, i.e., high symptom distress in the TICU, which decreased over time after admission to the thoracic ward, stable experience of person-centeredness and less restrictions in the fast-track group.

However, some results require further reflection such as the relatively high self-rated pain intensity score during the first six hours after extubation in the TICU. The patients reported a higher occurrence and intensity of pain in the TICU (89.9%, NRS medians of 4.5 and 4) than at the ward (75.0%, NRS median 2). Both units have a pain management strategy involving a combination of Acetaminophen and opioids (Oxycodone®) with the goal of achieving a pain intensity score of NRS ≤ 4. Inadequate perioperative analgesia activates the sympathetic nervous system, leading to other organ dysfunction, while pain-free patients exhibited reduced postoperative complications as well as length of hospital stay [3]. In ERAS^®^ protocols, adequate analgesia is of the utmost importance, as early mobilization and physiotherapy require an active and co-operative patient [3]. Although not investigated in this study, a clinical observation is that the chest tube is one major component that causes pain. After drainage removal, many patients reported substantially less thoracic pain.

Postoperative pain among cardiac patients is often undertreated, which is a risk factor for chronic pain, where women tend to have more problems than men [16]. In this study, no gender differences could be found in pain experience during the initial hospital stay, although the pain trajectory differed in women and men.

During the pre-operative phase, nurses at the surgical ward focus on providing post-operative pain management information to the patients, as suggested by Engelman et al. [4] but more effective strategies may be necessary. In ERAS^®^ protocols, patient information goes beyond the traditional form and is considered an educative measure to improve psychological readiness for surgery [3,4]. This entails discussions about the expected recovery as well as the patient´s active role in the process aimed at reducing the knowledge imbalance. The information also serves as a motivation for the patient and has been shown to reduce fear and post-operative analgesic use, which might improve postoperative recovery [3]. In line with this, the present study demonstrated that patients who experience pre-operative anxiety and worry also self-rated their experience of pain higher post-operatively, which is why patient education should be a perioperative area of focus.

Apart from pain, the three most frequently occurring symptoms in the TICU were also the most distressing ones. Feeling fatigued, being dependent on help from others and experiencing restrictions in physical activity are symptoms of being in a different state, making the patient feel exposed and vulnerable. This constitutes a severe intrusion into a patient’s autonomy [3]. Although expected from a healthcare perspective, inability to manage basic self-care due to tubes, catheters and restrictions is nevertheless a stressor for the patient that needs to be addressed. In the ERAS^®^ protocol, a central aspect is supporting the patient to regain autonomy as soon as possible [3].

At the surgical ward, pain was rated number six out of the ten most common symptoms and was not among the ten most distressing symptoms in the whole group. The focus on pain-management might explain the result, as well as the possibility that due to the study, pain was assessed more often, leading to enhanced pain management strategies. The most distressing symptom in the surgical ward was restrictions in social life. The fact that data were collected during the Covid-19 pandemic when the clinic had restrictions on visiting hours might have contributed to this distress.

It should be noted that the most common symptoms are not always experienced as the most distressing ones, which has been demonstrated in previous studies [17]. Patient-reported outcome measurements (PROM) are defined as measurements of the patient’s physical and psychosocial well-being such as pain, nausea and anxiety [18,19]. The inclusion of the patient perspective contributes to shared decision making and patient-centred care [20].

In the TICU, low or moderate correlations were found between time on the ventilator and fatigue and muscle weakness. In the surgical ward, a moderate correlation between time on the ventilator and concentration difficulties was observed. This indicates that longer ventilator treatment might predispose patients to higher symptom-distress in terms of fatigue and muscle weakness in the TICU and concentration difficulties at the surgical ward.

The experience of dizziness in the TICU and the surgical ward was a common symptom also reported in other studies [21]. This could be due to changes in baroreceptor function or medications such as beta-adrenergic blockers resulting in hypotension or bradycardia. Although not reported as causing high symptom distress by the patients in this study, the high frequency in the TICU (67.9%) should be addressed due to the risk of fall injuries or cardiovascular complications.

The participants reported that the care had been person-centred, irrespective of receiving conventional post-operative care or assigned to the fast-track protocol. This might be a result of many years of dedicated work to provide structured and sustainable person-centred care. To minimize any leverage or pressure from healthcare professionals regarding patients’ experience of person-centeredness, the questionnaire was completed at discharge and where possible, without any healthcare professional present.

### Limitations

Due to the study design no power calculation was performed. The sample size was pragmatic due to the circumstances described above. The non-randomized design of the study and small sample in the fast-track group limits the ability to draw firm conclusions, thus comparisons between the two groups should be interpreted with caution. The small sample size may limit generalization of the study findings. Furthermore, the implementation of a new fast-track programme might have contributed to bias in favour of the fast-track group, which received special attention from healthcare professionals.

The difficulties for patients to self-rate their pain immediately after extubation and when under the influence of sedative drugs should be taken into consideration when interpreting the high NRS medians and percentiles. Additionally, an inherent challenge with the instrument is measuring pain quantitatively. One limitation is that no further tests of validity or reliability for the BTSQ were conducted after the modification to fit more or less sedated patients in the TICU, thus the result should be interpreted with caution.

Finally, this study was carried out in a real-world clinical setting during the Covid-19 pandemic, resulting in practical inclusion difficulties as well as challenging circumstances in terms of implementing the new fast-track programme.

## Conclusions and implications

The conclusion of this study is that from a patient perspective a fast-track programme is equivalent to conventional care in terms of perceived symptom-distress and person-centeredness as well as reducing the length of ICU-stay. A reduction in the length of ICU-stay and subsequently overall hospital stay without increased suffering is a favourable outcome in the strive to restore patient autonomy and promote health.

The study provides detailed information on symptom distress after cardiac surgery, which could serve as an evidence-based foundation for symptom management. Areas in need of interventions that are highly suitable for standardized care plans enabling targeted strategies to relieve symptom distress have been identified. The relatively high pain intensity scores in the TICU need to be addressed, as do other common symptoms such as muscle weakness and fatigue. Feeling fatigued, being dependent on help from others and experiencing restrictions in physical activity in the TICU are symptoms experienced as highly distressing as they hamper autonomy. Limited autonomy is inherent in surgical care, thus normal and expected by healthcare professionals. However, for patients the lack of autonomy in the high-tech environment might be overwhelming. Therefore, an attentive and supportive approach is essential during the postoperative trajectory as well as a proper symptom assessment at the beginning of each shift.

The results contribute to an understanding of expected symptom patterns during cardiac surgery, which is of importance when healthcare personnel inform and educate patients and their family members perioperatively. An awareness of which symptoms are part of the recovery process prepares the patient and might increase self-efficacy. Future long-term studies are of the utmost importance to explore which symptoms remain and how patients recover from cardiac surgery.
